# Language exposure during infancy is negatively associated with white matter microstructure in the arcuate fasciculus

**DOI:** 10.1016/j.dcn.2023.101240

**Published:** 2023-04-11

**Authors:** Katiana A. Estrada, Sharnya Govindaraj, Hervé Abdi, Luke E. Moraglia, Jason J. Wolff, Shoba Sreenath Meera, Stephen R. Dager, Robert C. McKinstry, Martin A. Styner, Lonnie Zwaigenbaum, Joseph Piven, Meghan R. Swanson, for the IBIS Network

**Affiliations:** a Department of Psychological Sciences, Purdue University, West Lafayette, IN 47906, USA; b Department of Psychology, The University of Texas at Dallas, Richardson, TX 75080, USA; c Department of Educational Psychology, University of Minnesota, Minneapolis, MN 55455, USA; d Department of Speech Pathology and Audiology, National Institute of Mental Health and Neuro-Sciences (NIMHANS), Bangalore, India; e Department of Radiology, University of Washington, Seattle, WA 98195, USA; f Mallinckrodt Institute of Radiology, Washington University School of Medicine, St. Louis, MO 63130, USA; g Department of Psychiatry, University of North Carolina, Chapel Hill, NC 27599, USA; h Department of Pediatrics, University of Alberta, Edmonton AB T6G 2R3, Canada

**Keywords:** Home language environment, Arcuate fasciculus, Language development, Infancy, Diffusion tensor imaging

## Abstract

Decades of research have established that the home language environment, especially quality of caregiver speech, supports language acquisition during infancy. However, the neural mechanisms behind this phenomenon remain under studied. In the current study, we examined associations between the home language environment and structural coherence of white matter tracts in 52 typically developing infants from English speaking homes in a western society. Infants participated in at least one MRI brain scan when they were 3, 6, 12, and/or 24 months old. Home language recordings were collected when infants were 9 and/or 15 months old. General linear regression models indicated that infants who heard the most adult words and participated in the most conversational turns at 9 months of age also had the lowest fractional anisotropy in the left posterior parieto-temporal arcuate fasciculus at 24 months. Similarly, infants who vocalized the most at 9 months also had the lowest fractional anisotropy in the same tract at 6 months of age. This is one of the first studies to report significant associations between caregiver speech collected in the home and white matter structural coherence in the infant brain. The results are in line with prior work showing that protracted white matter development during infancy confers a cognitive advantage.

## 1 Introduction

The first two years of life are marked by dramatic gains in language acquisition. These gains are supported in part by the home language environment, specifically how much and the manner in which caregivers speak to their children (Hart and Risley, 1995, Huttenlocher et al., 1991, Huttenlocher et al., 2010, Swanson et al., 2019, Zimmerman et al., 2009). Recent studies have emphasized that the quality of the home language environment (e.g., vocabulary diversity, narrative language, joint attention, fluency, grammatical complexity, wh- question use) is pivotal for supporting children’s productive language development (Hirsh-Pasek et al., 2015, Rowe, 2012, Vernon‐Feagans et al., 2019). One aspect of caregiver speech quality that has received attention is conversational turn taking between caregivers and infants (Ferjan Ramírez et al., 2021, Romeo et al., 2018a, Romeo et al., 2018b, Swanson et al., 2019, Tamis-LeMonda et al., 2014). These back-and-forth communicative interchanges are thought to optimally support early language acquisition because they are time-sensitive (e.g., the parent responds quickly to an infant’s vocalization) and often responsive (e.g., the parent talks about what the child is looking at). Despite the growing literature demonstrating the intricacies of the link between the home language environment and language production, the neural mechanisms behind this association during infancy remain largely unknown.

Traditional models of language neurobiology focus on the arcuate fasciculus, or dorsal pathway, a tract which connects Broca’s area in the frontal lobe and Wernicke’s area in the posterior superior temporal gyrus. The arcuate fasciculus can itself be separated into three distinct segments: 1) the direct segment which provides a continuous connection between Broca’s and Wernicke’s areas, 2) the anterior indirect segment which connects Broca’s area to the inferior parietal cortex, and 3) the posterior indirect segment which connects the inferior parietal cortex to Wernicke’s area (Catani et al., 2004, Forkel et al., 2020). The arcuate fasciculus has been implicated in mapping speech sounds to articulatory, motor representations (Catani and Mesulam, 2008, Dick and Tremblay, 2012). The traditional model has been expanded upon to include a ventral pathway responsible for processing speech sounds to semantic meaning (Catani and Mesulam, 2008, Hickok and Poeppel, 2004; Saur et al., 2008). The ventral pathway is thought to primarily connect regions of the temporal lobe to the inferior frontal gyrus via several different white matter tracts: the uncinate fasciculus, the inferior fronto-occipital fasciculus, and the inferior and middle longitudinal fasciculi (Dick and Tremblay, 2012, Saur et al., 2008).

While traditional models of language neurobiology are based on the adult brain, some studies have shown that infants have “adult-like” language networks (Dubois et al., 2015, Silver et al., 2020). Dubois and colleagues demonstrated that infants scanned shortly after birth displayed a clear separation between the dorsal and ventral pathway tracts, as well as leftward asymmetry in the arcuate fasciculus (Dubois et al., 2015). Given the similarities between the language networks in adulthood and infancy, recent research has sought to investigate the associations between specific white matter pathways in infants and cognitive skills. Several studies have revealed a clear link between language-related tracts, including the arcuate fasciculus, and language skills in infancy (Girault et al., 2018; Sket et al., 2019). However, it does not appear that these results reflect a specialized network, but rather reflect global associations between white matter microstructure and general cognitive skill across multiple domains (Feng et al., 2019; Girault et al., 2018).

To date, investigations aiming to understand the neurobiological mechanism by which caregiver speech supports child language skills have been largely focused on functional brain development. For example, studies have reported negative associations between language input and resting state activation and functional connectivity during infancy (Brito et al., 2020, King et al., 2020). Investigations into these relations in older, school-aged, children have shown that conversational turn taking is positively associated with language related brain structure and function (Merz et al., 2019, Romeo et al., 2018a, Romeo et al., 2018b). With regard to white matter microstructure, Romeo and colleagues found that conversational turn taking was positively associated with structural coherence in the left arcuate fasciculus and superior longitudinal fasciculus in 4–6-year-old children. The significant associations shown between white matter tracts and measures of language input were significant only in language-related tracts (Romeo et al., 2018a). Contrary findings regarding the directionality of these brain-behavior relations indicate that further research is needed to disentangle how neural processes underlie language skills at different periods across development.

The current exploratory study of typically developing infants aimed to investigate the associations between the home language environment, at 9 and 15 months (9-mo and 15-mo henceforth, respectively), and white matter microstructure, at 3, 6, 12, and 24 months of age (3-mo, 6-mo, 12-mo, and 24-mo henceforth, respectively). The following tracts were examined: arcuate fasciculus (left direct fronto-temporal [direct segment], left posterior parietal-temporal [posterior indirect segment], right anterior fronto-parietal, inferior frontal-occipital fasciculus, inferior longitudinal fasciculus, splenium of the corpus callosum, and uncinate fasciculus, and corticospinal tract (as a control tract). Given the inconsistencies in the previous literature, the current study took an exploratory approach. We examined the home language environment for associations with both concurrent white matter microstructure and the development of white matter microstructure in language production-related white matter tracts. Results from this study could inform future work on the potential mechanistic role that white matter plays in how caregiver speech supports language production skills during infancy.

## 2 Methods

### 2.1 Participants

The present study included fifty-two infants from the Infant Brain Imaging Study (IBIS). IBIS is a prospective longitudinal study conducted at four clinical data collection sites: University of North Carolina at Chapel Hill (UNC), the Children’s Hospital of Philadelphia (PHI), University of Washington (SEA), and Washington University in St. Louis (STL). Data coordination was provided by the Montreal Neurological Institute at McGill University using the LORIS data management system (Das et al., 2016), and data processing of diffusion data was performed at the University of North Carolina at Chapel Hill. The procedures for this study were approved by local Institutional Review Boards at each data collection site, and written informed consent was provided by the infants’ parent or guardian prior to participation.

IBIS includes infants determined to have (based on family history) either a low- or high-likelihood for autism spectrum disorder (ASD). Infants with a high-likelihood for ASD had at least one older sibling with autism, therefore, approximately a 20% recurrence likelihood of ASD (Ozonoff et al., 2011) Infants with a low-likelihood for ASD had at least one typically-developing older sibling, and no first-degree relatives with a history of ASD or intellectual disability (Estes et al., 2015). To maximize statistical power, both infants with a low-likelihood and high-likelihood for ASD were combined into a single group for analyses and included in the current study. Infants with a high-likelihood for ASD were included in the present study only if they met criteria for typical development at 24 months (see Section **Typical Development Status**).

Infants were included in this study if they contributed at least one home-language recording at either 9 or 15 months (> 8 h in duration), at least one diffusion-weighted MRI scan at 3, 6, 12, or 24 months, and completed behavioral and diagnostic assessments at 24 months. All infants were determined to be typically developing (see Section **Typical Development Status**).

### 2.2 Behavioral Assessments

The Mullen Scales of Early Learning (MSEL) is a standardized assessment of cognitive development normed for children from birth to 68 months (Mullen, 1995). The MSEL was administered to infants at 24 months of age, and five subscale scores (visual reception, fine motor, gross motor, receptive language, and expressive language) were calculated in addition to the Early Learning Composite score (MSEL ELC) and Verbal Developmental Quotient (MSEL VDQ, Stephens et al., 2018). MSEL *t*-scores were used to determine whether infants were typically developing (see **Typical Development Status**). The MSEL ELC and MSEL VDQ are provided to characterize participant language skill (Table 1). Characteristic behaviors of ASD were assessed via the Autism Diagnostic Observation Schedule (ADOS; Lord et al., 2002) and Autism Diagnostic Interview-Revised (ADI-R; Lord et al., 1994) at 24 months. The Vineland Adaptive Behavior Scales II (Sparrow et al., 2005) was administered at 24 months to assess adaptive skills.

**Table 1:** Descriptive data for sample by groups (Low and high likelihood for ASD).

Variable	LL-neg (*N* = 25)	HL-neg (*N* = 27)	Test statistics
Age at scan [Mean (SD)]			
3-mo (*n* = 21)	3.4 (0.49)	3.4 (0.34)	*t* = −0.029, *p* = 0.977
6-mo (*n* = 34)	6.7 (0.74)	6.4 (0.36)	*t* = 1.48, *p* = 0.151
12-mo (*n* = 19)	12.2 (0.49)	12.6 (0.36)	*t* = −1.863, *p* = 0.092
24-mo (*n* = 22)	25.7 (2.41)	24.4 (0.37)	*t* = 1.690, *p* = 0.128
			
Age at LENA [mean (SD)]			
9-month	9.7 (0.56)	9.7 (0.60)	*t* = −0.305, *p* = 0.762
15-month	15.6 (0.67)	15.7 (0.72)	*t* = −0.322, *p* = 0.749
			
Males within likelihood group (%)	60	62.3	χ^2^ = 0.125, *p* = 0.724
			
Mullen VDQ [Mean (SD)]	110.38 (11.95)	107.53(15.61)	*t* = 1.063, *p* = 0.293
Mullen ELC [Mean (SD)]	113.72(11.95)	108 (15.06)	*t* = 1.523, *p* = 0.134
			
Race within likelihood group (%)			χ^2^ = 1.601, *p* = 0.659
White	72	77.8	
Asian	0	3.7	
African-American	8	3.7	
More than one race	20	14.8	
			
Maternal education within likelihood group (%)			χ^2^ = 6.837, *p* = 0.145
High school/some college	12	29.6	
College degree	40	44.4	
Some grad school/grad degree	48	26	

### 2.3 Typical development status

All infants in the current study were determined to be typically developing. Infants at a high-likelihood for ASD met the following criteria for typical development (Chawarska et al., 2016): (a) ADOS calibrated severity score ≤ 2, (b) no more than one MSEL subtest *t*-score < 35, (c) no MSEL subtest *t-*score < 30, and (d) no clinical best estimate diagnosis of ASD at 24 months. Clinical best-estimate diagnoses were made by licensed clinicians based on DSM-IV-TR criteria using all available assessment data including the ADOS, ADI-R, MSEL, and the Vineland Adaptive Behavior Scales II.

### 2.4 Home language recordings

Day long audio recordings were collected when infants were 9 and/or 15 months of age. Infants wore a small recording device (LENA Research Foundation Digital Language Recorder) designed to capture up to 16 h of home language audio data. Families were instructed to record two full days of audio, turning on the recorder when the infant woke up, and leaving it on throughout the day and into the night. Full recording procedures are available in Swanson et al. (2019).

Software automatically extracted infant and adult vocalizations from the home language recordings (LENA Pro software suite V3.3.4; Xu et al., 2009). Primary validation analysis of the LENA software indicated 82% accuracy for adult vocalization and 76% for child vocalizations (Xu et al., 2009). Recent analyses have reported similar correlations between LENA’s automatic measures and human annotation (Merz et al., 2019; Soderstrom et al., 2013).

Given the exploratory nature of the current study, each LENA derived measure (Adult Word Count [AWC], Child Vocalization Count [CVC], and Conversational Turn Count [CTC]) were extracted via LENA software for further analysis. AWC is the number of words spoken by an adult near the infant wearing the recorder. CVC is the number of speech-like vocalizations produced by the infant wearing the recorder. Speech-like vocalizations produced by an infant can include words, babbles, or pre-speech communicative sounds (e.g., squeals, growls, and raspberries; Oller, 2000). CTC is the number of reciprocal verbal exchanges between the infant wearing the recorder and a nearby adult occurring within 5 s of each other. The aforementioned variables were summed across each recording day, and then averaged across the two-recording days to produce daily average counts for the 9 and/or 15-month time points. Previous literature—from which the current data is a subset—indicated that there was no significant difference between the two-recording days at either 9 or 15 months (Swanson et al., 2019).

### 2.5 Image acquisition

Neuroimaging was conducted at each of the data collection sites using identical 3 T Siemens TIM Trio scanners (Siemens Medical Solutions, Malvern, PA.) equipped with 12-channel head coils. Scans were collected during natural sleep. DWI scans were acquired using an ep2d_diff sequence with a field of view of 190 mm (3, 6, and 12 months) or 209 mm (24 months), 75–81 transversal slices, slice thickness of 2 mm isotropic, 2 ` 2 ` 2 mm^3^ voxel resolution, TR = 1 2800–13,00 ms, TE = 102 ms, variable b-values from 0 to 1000 s/mm^2^ in increments of 40, 25 gradient directions, and a scan time of 5–6 min. One *b* = 0 image was collected during image acquisition. Scanner reliability was assessed annually via the use of both geometric and human phantoms.

### 2.6 Image preprocessing

Both automated and manual quality control measures were implemented to correct for infant motion and common artifacts in all generated diffusion weighted images (DWI). DTIPrep (Oguz et al., 2014) was utilized to 1) automatically correct for motion and eddy current artifacts, and 2) to detect and remove DWIs with artifacts. After automated quality control procedures, expert raters manually removed DWIs with remaining artifacts. To prevent the inclusion of datasets with a low signal-to-noise ratio, datasets were excluded from analysis if they had less than 18 of 25 remaining gradient DWIs. Diffusion tensor images (DTI) were generated via weighted least square estimation and zeroing of tensors with negative Eigenvalues.

### 2.7 Computational anatomy mapping

DTI images were co-registered to create an unbiased DTI atlas using DTI-AtlasBuilder. First, DTI images were affinely registered to a previously generated infant T2 weighted template. Next, an unbiased diffeomorphic atlas was created via affine, followed by deformable diffeomorphic registration. The displacement fields created in this procedure mapped the study-specific atlas space to individual subject spaces through a nonlinear, invertible transformation. Tensor maps were transformed into the atlas space and averaged to produce the final DTI atlas (Short et al., 2022, Verde et al., 2014). In this study, each infant’s DTI data was processed as described above and mapped into the final DTI atlas, which served as a reference space for tractography and tract parameterization (Verde et al., 2014, Swanson et al., 2018).

### 2.8 Fiber tractography

3D Slicer (www.slicer.org) was used to create seed label maps in the DTI atlas. Further post-processing based on known fiber tract anatomy was performed using FiberViewerLight (http://www.ia.unc.edu/dev/) to further delineate fibers of interest from erroneous fibers. Label map tractography was conducted for each white matter tract. Label map tractography of the arcuate fasciculus yielded two segments for the left arcuate (direct fronto-temporal and posterior parietal-temporal) and one segment for the right arcuate (anterior fronto-parietal). The arcuate is highly variable in humans and is late to myelinate; given these two factors, quantitative fiber tracking in the atlas space may result in asymmetric segmentations of the arcuate. The corpus callosum was segmented based on the Hofer and Frahm (2006) anatomical parcellation method. This method yields corpus callosum segments that project to prefrontal regions (Section I, i.e., genu), the premotor and supplementary motor cortex (Section II), the primary motor cortex (Section III), the somatosensory cortex (Section IV), and the parietal, temporal, and occipital regions (Section Vb, i.e., splenium).

Fractional Anisotropy (FA) is a measure of the degree of anisotropy along a given axon with values ranging from 0 to 1; a value of 0 indicates isotropic diffusion, while a value of 1 is indicative of anisotropic diffusion, and highly organized axonal structure. Average FA values were generated across each of the following bilateral tracts: corticospinal tract, inferior frontal-occipital fasciculus, inferior longitudinal fasciculus, and uncinate fasciculus. FA values were generated across the remaining tracts: left direct fronto-temporal arcuate fasciculus, left posterior parieto-temporal arcuate fasciculus, right anterior fronto-parietal arcuate fasciculus, and splenium of the corpus callosum. Other DTI measures were also studied, including radial diffusivity (RD), a measure of diffusivity perpendicular to axonal fibers, and axial diffusivity (AD), a measure of diffusion parallel to fiber tracts.

### 2.9 Statistical analysis

As of April 26th, 2021, data were available for 52 infants who completed at least one DTI scan and one home-language recording (low-likelihood, *N* = 25, high-likelihood, *N* = 27). All analyses were performed using R, version 4.1.1 (R Core Team, 2021).

To optimize power and identify potential covariates impacting FA measurements we used principal component analysis (PCA, see Abdi and Williams, 2010). To analyze a set of variables measured on the same observations, PCA creates a set of new orthogonal variables (called principal components)—computed as linear combinations of the original variables—that are maximally correlated with the original variables. In PCA, the components are ordered by their variance (called an *eigenvalue*). This multivariate statistical approach is particularly useful when the original dataset contains inter-correlated variables (as in here), because, in this case, only a few principal components can extract most of the variability in the dataset.

**Fig.1:**
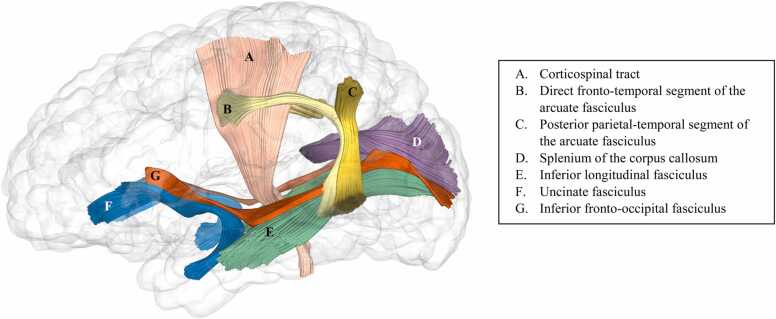
Glass brain image with a priori tracts of interest.

The time point with the highest number of participants (6-mo, *N* = 34) was selected for PCA and a data matrix was created with the rows being participants and columns being FA values of the a priori tracts of interest (*P* = 14). The analysis was performed using the epPCA and epPCA.inference.battery functions from the ExPosition and InPosition R-packages (Beaton et al., 2014). The columns of the data table were centered and normalized prior to the analysis. The number of components to keep was determined by running a permutation test: In this test, the values of each variable are permuted, a PCA is performed and the eigenvalues of the components are stored; the procedure is repeated many times (here, 1000). The probability associated to a given component is estimated by the proportion of permuted eigenvalues larger than its eigenvalue. Here, only two components showed a *p*-value smaller than.05 and were kept for further analysis (see scree plot in Fig. 1, Supplement).

**Fig. 2:**
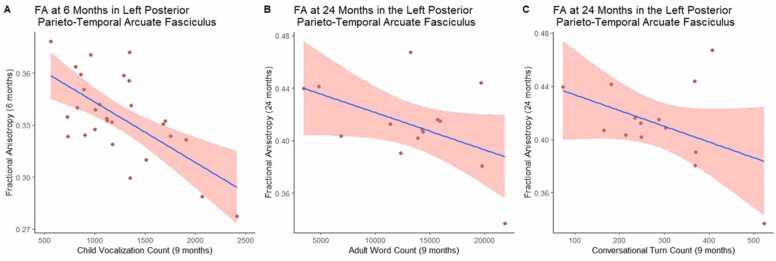
SScatterplots of language environment variables and FA with line of best fit.

To interpret the principal components, scatterplots were generated with the values, respectively, of Component 1 for the horizontal axis and Component 2 for the vertical axis. In these component plots, one point represents one participant and the distance between participants represents their similarity. All our covariates were nominal variables (e.g., data collection sites with four groups). To evaluate the effect of one variable, the mean of each group of this variable was computed (and projected on the component plots) and a 95% confidence interval of each mean computed using a bootstrap resampling procedure (see, Abdi et al., 2009; Le Roux and Rouanet, 2011) was drawn on the plots. On these plots, when the confidence intervals of two groups overlap, these two groups cannot be considered significatively different (at the α = .05 level); by contrast, when the confidence intervals do *not* overlap, the groups differ at the α = .05 level (Abdi et al., 2009). The following covariates were tested with this procedure (see, Fig. 2, Supplement): sex of the infant (Male/Female), maternal education level (High School Degree or Some College/College Degree/Graduate Degree), data collection site (UNC/STL/SEA/PHI), and infant’s likelihood for ASD (low-likelihood/high-likelihood).

FA values for all tracts were determined to approximate a normal distribution via visual inspection of histograms. Thus, classic regression models were used to examine the cross-sectional associations between FA values at 3, 6, 12, and 24 months and home language environment variables at 9 and 15 months separately. Participant’s age at MRI scan was selected a priori to be included as a covariate in all models. False Discovery Rate (FDR) corrections were implemented for all tests conducted using the same response variable to correct for multiple comparisons using the one-step model described by Benjamini and Hochberg (1995). FDR corrected *p*-values are presented as *q*-values. Significant models were followed up with tests of convergent validity using AD and RD values and tests to examine associations between home language recording variables and individual arc lengths along the fiber tracts.

Mixed models were used to model the longitudinal association between FA and the home language environment across time. All models had longitudinal FA as the response variable with home language environment variables serving as the fixed effect. Participant identification number was included as a random intercept in all longitudinal models.

## 3 Results

### 3.1 Participant characteristics

Participant demographic information and cognitive assessment scores are presented in Table 1. While low-likelihood and high-likelihood participants were combined into a single group in all following analyses, group differences on descriptive measures can be found in Table 1; none of the tests were found to be significant. One hundred and seventy-four home language environment recording days were collected from 52 participants using home language recorders, with 97.70% (*N* = 170) recording days having full 16 h of data, and the remainder having between 8- and 16-hour recordings. The shorter recordings were generally a consequence of the parents turning the recorder off during bedtime and hence the data was not adjusted for recording time (Swanson et al., 2019). Table 2 shows the Pearson’s product-moment correlation between the home language recording variables at different time points.

**Table 2:** **Means, standard deviations, and Pearson’s correlations with confidence intervals**.

**Variable**	***M***	***SD***	**1**	**2**	**3**	**4**	**5**
							
1. AWC 9-mo	15546.63	6441.34					
							
2. AWC 15-mo	13752.49	6049.75	.87 * *				
			[.77,.93]				
							
3. CVC 09-mo	1215.83	401.87	-.07	-.01			
			[− .37,.25]	[− .34,.32]			
							
4. CVC 15-mo	1293.38	387.78	.32	.44 * *	.18		
			[− .01,.58]	[.18,.65]	[− .16,.48]		
							
5. CTC 9-mo	300.90	100.50	.58 * *	.61 * *	.61 * *	.46 * *	
			[.33,.75]	[.36,.78]	[.37,.77]	[.15,.68]	
							
6. CTC 15-mo	339.96	137.97	.66 * *	.79 * *	-.05	.75 * *	.60 * *
			[.42,.81]	[.66,.88]	[− .37,.28]	[.59,.85]	[.34,.78]
							

*Note. M* and *SD* are used to represent mean and standard deviation, respectively. Values in square brackets indicate the 95% confidence interval for each correlation. * indicates *p* < .05. * * indicates *p* < .01.

The PCA procedure identified “data collection site” as the only potential covariate having at least two groups differing from each other, and so, “data collection site” was kept as a covariate for the follow-up analyses.

#### 3.1.1 Associations between the language environment and white matter FA

General linear multiple regression models were used to determine if there was a significant association between home language recording variables and FA values at 3-, 6-, 12-, and 24-month timepoints. Separate models were run for home language recording variables at 9 and 15 months. Forty-one and 47 participants had home language recording data at 9 and 15 months, respectively.

Results (Table 3) showed a significant negative association between CVC at 9 months and FA in the left posterior parieto-temporal arcuate at 6 months (*q* =.012), indicating that infants who produced more speech-like vocalizations had less coherent white matter organization. Significant negative associations were also found between AWC and CTC at 9 months and FA in the left posterior parieto-temporal arcuate at 24 months (*q* =.013, *q* =.012, respectively). Fig. 2 shows the scatterplots with line of best fit between the language environment variables and FA. Convergent associations (Table 3) were found between AWC and CTC at 9 months and RD in the left posterior parieto-temporal arcuate at 24 months (*q* =.007, *q* =.004, respectively). The remaining RD models did not yield statistically significant results. We also examined associations between AD and home language recording variables, but none of the models were significant prior to FDR correction.

**Table 3:** Significant associations between the language environment and DTI measures (FA & RD) of the left posterior parieto-temporal arcuate fasciculus.

DTI measure		Visit label (MRI scan)	Variable	*F*	*p*	*q*	*R*^*2*^	95% CI
FA		6-mo	CVC at 9 months	14.286	.001 * *	.012 *	.449	.244 –.653
			Age at scan	.008	.932			
			Site	.414	.745			
		24-mo	AWC at 9 months	22.506	.001 * *	.013 *	.797	.686 –.908
			Age at scan	15.776	.003 * *	.029 *		
			Site	4.357	.047 *	.272		
		24-mo	CTC at 9 months	23.802	.001 * *	.012 *	.805	.698 –.912
			Age at scan	15.482	.003 * *	.020 *		
			Site	6.841	.016 *	.126		
RD		24-mo	AWC at 9 months	26.812	.001 * **	.007 * *	.825	.727 –.922
			Age at scan	29.698	< .001 * **	.005 * *		
			Site	.369	.701			
		24-mo	CTC at 9 months	31.078	< .001 * **	.004 * *	.843	.755 –.931
			Age at scan	32.176	< .001 * **	.004 * *		
			Site		1.581	0.258		

**Fig.3:**
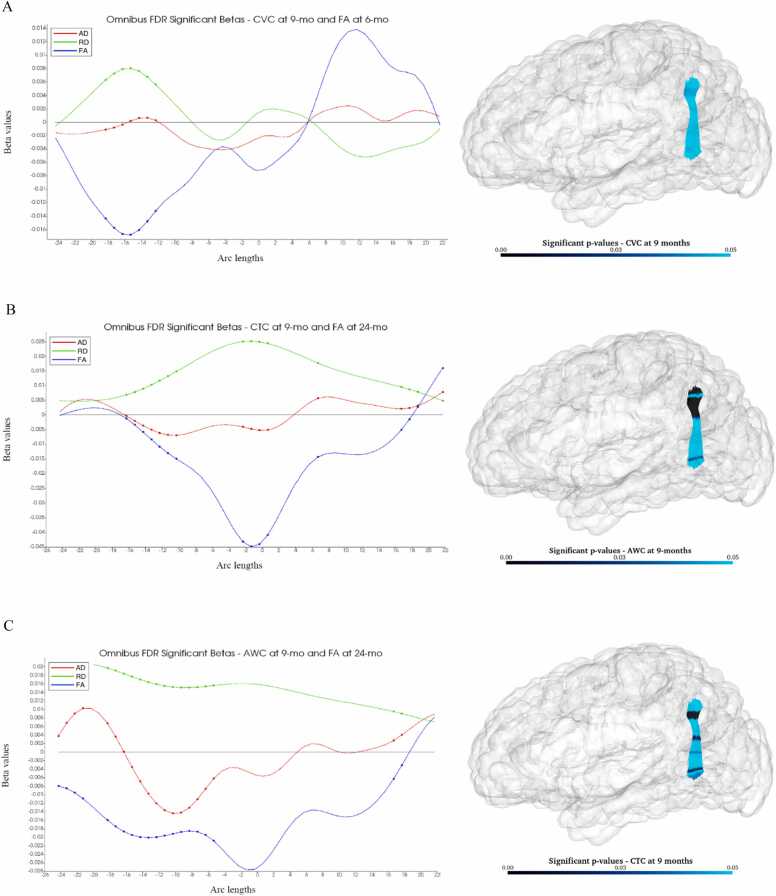
Visualization of significant associations on different arc lengths of the left posteriori parieto-temporal arcuate fasciculus generated using Slicer. 3 A. Visualization of CVC at 9 months and 6-month FA of the left posterior parieto-temporal arcuate fasciculus 3B. Visualization of AWC at 9 months and 24-month FA of the left posterior parieto-temporal arcuate fasciculus 3 C. Visualization of CTC at 9 months and 24-month FA of the left posterior parieto-temporal arcuate fasciculus.

We conducted follow up analyses to explore associations between the home language recording variables and individual arc lengths of the left posterior parieto-temporal arcuate fasciculus. Significant *p*-values along the arc lengths were generated using the FADTTS (Functional Analysis of Diffusion Tensor Tract Statistics) toolbox and its corresponding graphical user interface, FADTTSter (Noel et al., 2017). Fig. 3 presents the results. Omnibus tests using FA, RD, and AD were used to maximize power. On the plots, the black line is the significance level while the red, green and blue lines represent the variability that each DTI metric contributed to the model; the further the line is from the significance threshold, the greater the contribution to the model. The x-axis denotes the arc length along the tract while the y-axis contains the beta values of the (multivariate) generalized linear model with independent and dependent variables and covariates. The dots represent arc lengths with significant associations between the DTI metrics and the home language recording variables. Using MergeStatWithFiber (Verde et al., 2014), the *p*-values were mapped onto the corresponding location on the fiber tract. Darker regions correspond to the dots in the plot which show significant associations. The arc lengths that show significant associations between AWC and CTC at 9 months and the left posterior parieto-temporal arcuate FA at 24 months largely represent the superior part of the tract that has terminations in Geschwind’s territory.

##### 3.1.1.1 Associations between the language environment and white matter across development

**Fig. 4:**
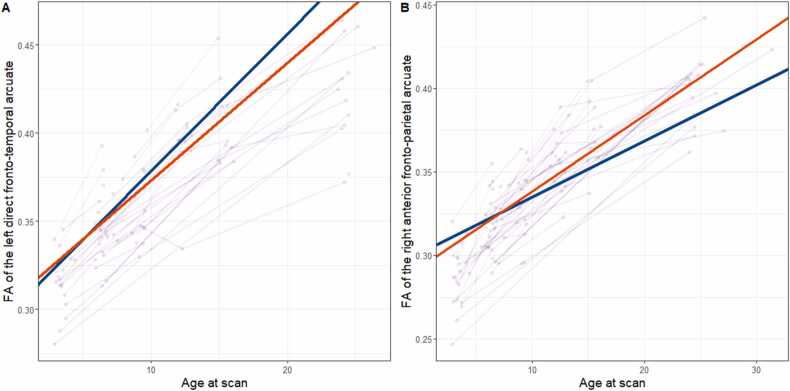
Spaghetti plots of FA development in relation to LENA variables. Purple lines indicate individual trajectories of FA development across time. Bold lines are for illustration only and represent FA development at one standard deviation below (blue line) and above the mean (red line) for conversational turn counts at 9 months (Panel A) or adult word counts at 15 months (Panel B).

Next, we investigated the association between home language recording variables and FA development from 3 months to 24 months. Nine participants had data at three time points, 26 had data at two time points, while 17 participants contributed data from only one time point. Linear mixed model results showed that the FA development in the left direct fronto-temporal arcuate was negatively associated with 9-month CTC (*p* = .021, *q* =.248) [Fig. 4A]. FA development in the right anterior fronto-parietal arcuate was positively associated with 15-month AWC, with the interaction between AWC and age being significant (*p* = .020, *q* =.234) [Fig. 4B]. However, none of these associations withstood FDR corrections for multiple comparisons.

## 4 Discussion

The results of the current study indicate that the home language environment has a direct association to white matter coherence during infancy. The home language environments of typically developing infants were measured when infants were 9 and 15 months, and white matter coherence was measured when infants were 3, 6, 12, and 24 months of age. Participants who vocalized the most at 9 months also had lower fractional anisotropy in the left posterior parieto-temporal arcuate fasciculus at 6 months. Similarly, participants who heard more adult words and engaged in more conversational turns at 9 months also had lower fractional anisotropy values in the left posterior parieto-temporal arcuate fasciculus measured at 24 months.

Previous literature has found that the home language environment is positively associated with language related brain structure in school-aged children (Merz et al., 2019, Romeo et al., 2018a). Romeo and colleagues (2018a) observed that children who engaged in more conversational turns had more coherent white matter organization in the left arcuate fasciculus. Additional analysis revealed that the home language environment was uniquely associated with left lateralized language related white matter tracts (i.e., arcuate fasciculus and superior longitudinal fasciculus). Likewise, the current study found significant associations between caregiver speech and white matter localized to the left hemisphere. However, contrary to these findings, the current study found that infants who produce more vocalizations, experience more adult speech, and participate in more conversational turns have lower FA values (and higher RD) in the left arcuate fasciculus. While the findings of our study are incongruous with research of school-aged children showing positive associations with FA (Romeo et al., 2018a), they are consistent with previous research of infants showing negative associations between caregiver speech and brain function (Brito et al., 2020; King et al., 2020). Differential patterns of results may be observed in infants and older children given the distinct neurobiological processes occurring across development.

The complex interplay between myelination and plasticity may provide a possible neurobiological explanation for the negative association between caregiver speech and white matter in the infant brain reported in the current study. Myelination is the process by which neuronal axons become sheathed in a lipid layer, which serves as an insulator, thereby increasing the speed of neuronal signal transmission (Aggarwal et al., 2011, Nave and Werner, 2014). The path to myelination is a prolonged process that begins prenatally and continues in a non-linear manner until early to middle adulthood. While there is current appreciation that myelination is activity-dependent and can change in response to learning and experience across the lifespan, more effort may be required to adaptatively “re-wire” a mature and established white matter network, and less effort to change an immature network. This is likely the case because myelination limits pathophysiological plasticity (McGee et al., 2005). Because a highly plastic brain may facilitate the development of complex cognitive skills such as language acquisition, in some contexts, a less myelinated network may be optimal. Indeed, research in studies of infants have shown that protracted white matter development is associated with better cognitive performance (Deoni et al., 2014, Girault et al., 2018). Results from the current study are in line with this previous work and further indicate that protracted myelination during infancy may confer unique benefits for cognitive development and language acquisition.

An alternative neurobiological explanation is related to axonal pruning. A large number of synapses are generated during development and later eliminated based on experience (Faust et al., 2021, Millán et al., 2018, Sakai, 2020). Axonal pruning is a regressive process that is essential for refining neural connections (Riccomagno and Kolodkin, 2015). RD as a measure is more sensitive to axon composition and density, than axial or medial diffusivity (Klawiter et al., 2011). The results of the current study could be reflecting that infants who experience more caregiver speech have arcuate fasciculi fiber tracts that are less dense due to experience dependent pruning.

### 4.1 Clinical implications

Thirty years of research have shown that infants and toddlers who hear the most caregiver speech have the best language skills (Hart and Risley, 1995, Huttenlocher et al., 1991, Huttenlocher et al., 2010, Swanson et al., 2019, Weisleder and Fernald, 2013). Recent work has indicated the importance of the quality of caregiver speech (e.g., reciprocity, vocabulary diversity, grammatical complexity) above sheer input quantity (Hirsh-Pasek et al., 2015, Rowe, 2012, Vernon‐Feagans et al., 2019). Research has also clearly demonstrated that early language skills are predictors for later development including school readiness and academic outcomes (Fiorentino and Howe, 2004, Pace et al., 2019). Given the unique role that language plays in development, caregiver speech has been a target of intervention studies (Feil et al., 2020, Leung et al., 2020) and public health campaigns (e.g., Too Small to Fail). Such programs have demonstrated effectiveness in changing the quantity and quality of caregiver speech directed towards children—an effect indicating that caregiver speech is a valid treatment target (Feil et al., 2020, Leung et al., 2020). The current study contributes to this body of research by revealing important information about timing; if caregiver speech at 9 months is associated with later brain development, the ideal timing to start an intervention may be when infants are quite young and still preverbal.

Large scale public policy campaigns to increase caregiver speech to infants were largely motivated by Hart and Risley’s finding that infants from low-income homes hear fewer words than infants from higher income homes. This “word gap” has been reported in other studies (Hoff, 2003; Huttenlocher et al., 2010; Rowe, 2017)), but some have highlighted the large amount of variation within social classes and disputed the actual size of the “word gap” (Sperry et al., 2018). Public health campaigns that focus solely on increasing caregiver speech are putting the impetus for change on the individual while overlooking the role that society and institutional practices play in creating disparities in infant development (for a review on how developmental science can be changed to upend racism see Buchanan et al., 2021). For example, caregivers that have extended paid leave policies have time at home with infants to bond and nurture strong attachment relationships without the financial stress of choosing between pay and time with the infant. However, in the United States there are inequities in the availability of paid leave—while there is no difference across ethnic/racial groups in the amount of leave taken, Black mothers are the most likely to receive no pay during leave and the least likely to receive full pay (Goodman et al., 2021). The authors highlight one important area of how unequal access to resources may impact caregiver speech and/or child language, but many others are relevant (e.g., quality and type of non-parental childcare, resource allocation to schools, residential segregation). The current study does not examine associations between socioeconomic status and amounts of caregiver speech, nor is the sample well suited for such an approach due to limitations in variation in socioeconomic status.

Intervention studies aiming to support or improve language skills in infants and toddlers are hampered by a lack of outcome measures that are objective and sensitive to change. The use of neurobiological monitoring biomarkers addresses these limitations as white matter metrics are dynamic and not subject to placebo effects (Swanson and Hazlett, 2019). For example, in a study of 8–10-year-old children who were poor readers, an intensive intervention resulted in more normative patterns of white matter in the left anterior centrum semiovale (Keller and Just, 2009). Importantly, the amount of fractional anisotropy change was associated with the amount of improvement in phonological decoding skills. The mechanism by which caregiver speech supports infant language skills is presumably by altering brain development. A foundational intervention study using white matter as a monitoring biomarker would provide support for this theoretical mechanism. A recent study of 4–6-year-old children is a notable example of such an approach; in the study parents who were assigned to the family-based intervention showed increases in parent-child conversational turns and parents who increased their conversational turn counts the most had children with the greatest increase in language scores (Romeo et al., 2021). Interestingly, there was no intervention effect for cortical thickness measures, but across all participants there was a significant association between changes in conversational turns and changes in cortical thickness (in the left supramarginal and inferior frontal gyri). Last, results from Romeo et al. (2021) provide initial evidence for the brain being a mechanism by which caregiver speech supports language by showing that changes in the supramarginal gyrus mediated the association between change in conversational turns and change in language skills.

### 4.2 Limitations

While automated measures of the home language environment offer the convenience of processing large amounts of data quickly, they are not devoid of limitations. The main limitation of this approach is the reliability of the automated tool to perform correct diarization of the data (e.g., annotate the child is speaking when the child is actually speaking). While initial validation results by the LENA Research Foundation indicated acceptable sensitivity of their software (82% for adult words and 76% for infant vocalizations; Xu et al., 2009), a recent systematic review reported large variability across studies in the correlation values between hand-coding and LENA Research Foundation estimates (*r* range = [.37 –.99]; Räsänen et al., 2019). As more automated processing tools become freely accessible and widely available (e.g., DiViMe; Le Franc et al., 2018), researchers should carefully consider which tool will yield the highest quality data for their specific dataset. We also acknowledge that many regression models did not yield significant results. The sample size was underpowered to detect small effects making Type II errors possible. A replication with a larger sample could reveal additional significant associations between caregiver speech and white matter tracts.

The participants of the current sample lack diversity in terms of race, ethnicity, and maternal education levels. As such, the external validity of the current findings is limited. Current efforts by the research team are addressing this limitation with an ongoing study. The current study only included western infants from English speaking homes. Culturally, there are differences in how caregivers communicate with their infants (Weber et al., 2017, Farran et al., 2016), and imposing western ideals on other cultures would be ethnocentric and could lead to misunderstanding of the study implications.

The data from the current study included some participants who had an older sibling with ASD and some who had a typically developing older sibling. Statistical analyses indicated that the status of having an older sibling with autism or not did not contribute meaningfully to the results. However, the sample size of infants with and without an older sibling with ASD was too small to conduct group comparisons. Further, this study was limited to studying language production. While the LENA system provides a detailed account of naturalistic language production, it does not allow for the analysis of language comprehension. Future work should investigate the potential associations between language comprehension and white matter microstructure during infancy.

Last, we studied infant brains using diffusion tensor imaging tractography—a non-invasive MRI technique which generates proxy measures of white matter microstructure. While DTI provides an estimate of white matter structural coherence, it does not indicate which attributes (e.g., myelination, axon density) contribute to the estimate. Additionally, regions of crossing fibers may result in areas of signal drop out due to poor model fit (Dubois et al., 2014). The current study used validated tractography approaches with anatomically informed regions using white matter atlases to ensure that the generated fiber tracts were anatomically relevant.

### 4.3 Future recommendations

Advancements in DTI since the collection of the current dataset include scan sequences that are efficient at handling crossing fibers which are known to make fiber tracking of infant temporal lobe tracts a particular challenge. One notable example, is High Angular Resolution Diffusion-Weighted Imaging (HARDI; Berman et al., 2013). HARDI sequences collect data on a larger number of gradient directions than DTI, allowing for the discrimination of crossing fibers within a given voxel. Using HARDI sequences for infant neuroimaging studies will likely provide a fuller representation of temporal lobe fiber tracts which may have important implications for the interpretation of results from brain-behavior analyses.

The current study adds to the body of research showing that caregiver speech is a protective factor for productive language development, because this study provides initial insights into potential structural brain mechanism in infants. However, the current sample is underpowered to determine how caregiver speech, infant brain development, and infant language skills are all interconnected across early development. Such integrated biobehavioral environmental approaches will be essential to a fuller understanding of how to best support infant brain and language development. For example, such approaches would yield important information on the timing of when caregiver speech has the greatest influence on brain development. Filling this gap in the literature would increase the effectiveness of interventions designed to support school readiness through right home language environments.

In conclusion, the current research study found that infants who heard more caregiver speech, exchanged more conversational turns, and used more speech-like vocalizations also had lower white matter coherence in language production-related white matter tracts. This research contributes to a small but growing body of infancy research showing that protracted white matter development is associated with better cognitive skills. The results of this study also contribute important information on developmental cascades; namely that the home language environment during *infancy* is associated with both brain and behavior in the *toddler years*. The timing of this developmental cascade is important for clinical and public health research that targets caregiver speech as a way to close the achievement gap and support kindergarten readiness. Providing supports for caregivers during the earliest months of life may results in optimal outcomes as infancy is a period of high brain plasticity and a sensitive period for language development.

## Declaration of Competing Interest

The authors declare that they have no known competing financial interests or personal relationships that could have appeared to influence the work reported in this paper.

## Data Availability

Core data collected as part of the Infant Brain Imaging Study is available through the NIMH data archive. Home language recordings are not publicly available since confidentiality could not be assured.
