# Supplementary material for: Language exposure during infancy is negatively associated with white matter microstructure in the arcuate fasciculus
Source: Dev Cogn Neurosci. 2023 Apr 11;61:101240. doi: 10.1016/j.dcn.2023.101240 (PMC10130606; doi:10.1016/j.dcn.2023.101240)
Supplement: Supplementary file 1 — Supplementary material. [file mmc1.docx]

**SUPPLEMENT**
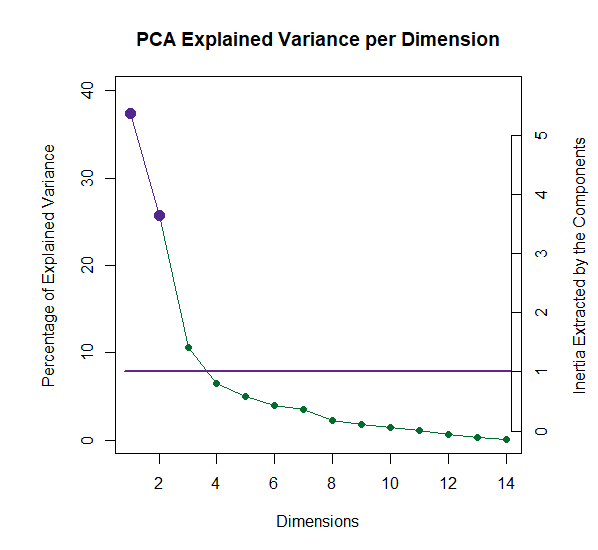


**Fig 1.**

**A B**


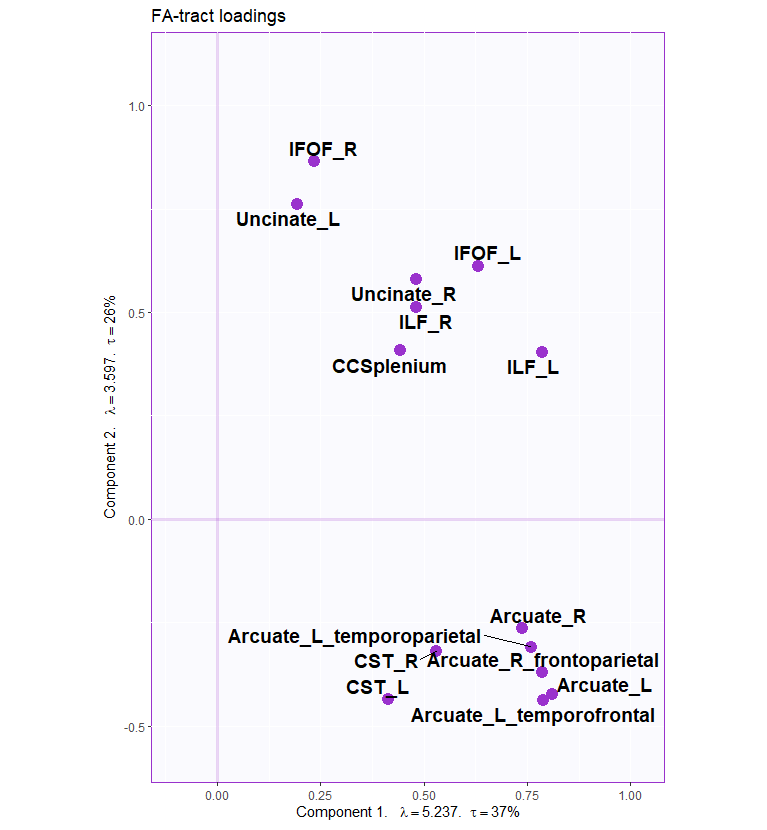


Fig. 1 panel A shows the scree plot used to determine the number of components to be examined using Principal Component Analyses. The purple line (“Kaiser” line) represents an eigenvalue equal to 1. Purple dots identify eigenvalues (and therefore components) significant at the α = .05 level using a permutation test.
Panel B plots the loadings for the tracts on dimensions 1 and 2 in the principal component space.


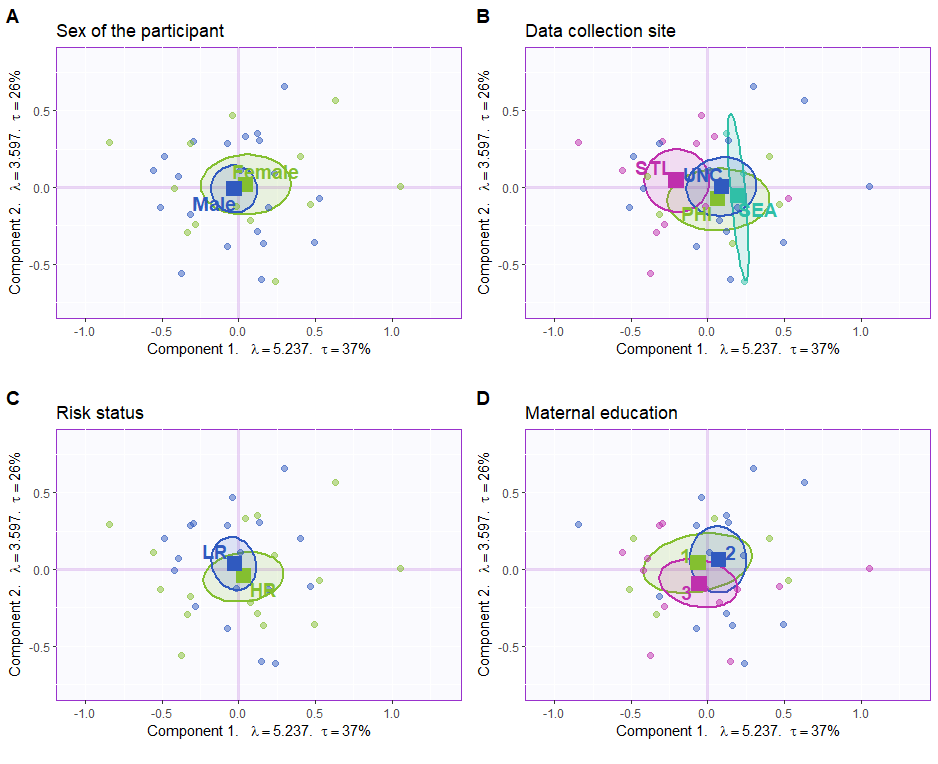
**Fig 2.**

Fig 2 shows the row factor score plot in dimension 1 and 2, of each covariate with the group means and bootstrapped 95% confidence intervals. Panel B shows non-overlapping levels of the “data collection site” variable, thereby adding unique variance to the dataset. Based on these results, data collection site was included as a covariate in all regression models.

The following tables contain results of regression models run using *a priori* tracts across various MRI and LENA data collection time points:

| Left Direct Fronto-Temporal Arcuate Fasciculus | | | | | | | | | | | | | |
| --- | --- | --- | --- | --- | --- | --- | --- | --- | --- | --- | --- | --- | --- |
|  |  | 3-month DTI | | | 6-month DTI | | | 12-month DTI | | | 24-month DTI | | |
| LENA Timepoint | Predictors | F | *p* | *q* | F | *p* | *q* | F | *p* | *q* | F | *p* | *q* |
| 9-month | AWC | 0.805 | 0.387 |  | 1.777 | 0.196 |  | 1.569 | 0.257 |  | 2.594 | 0.142 |  |
|  | Age | 2.693 | 0.127 |  | 1.441 | 0.242 |  | 0.804 | 0.404 |  | 1.263 | 0.290 |  |
|  | Site | 5.089 | 0.017* | 0.102 | 1.049 | 0.391 |  | 0.481 | 0.640 |  | 0.558 | 0.591 |  |
|  |  |  |  |  |  |  |  |  |  |  |  |  |  |
|  | CVC | 0.212 | 0.653 |  | 1.890 | 0.183 |  | 1.959 | 0.211 |  | 1.376 | 0.271 |  |
|  | Age | 3.016 | 0.108 |  | 0.575 | 0.456 |  | 1.259 | 0.305 |  | 0.286 | 0.606 |  |
|  | Site | 4.254 | 0.029* | 0.148 | 0.620 | 0.610 |  | 0.760 | 0.508 |  | 0.701 | 0.521 |  |
|  |  |  |  |  |  |  |  |  |  |  |  |  |  |
|  | CTC | 0.001 | 0.978 |  | 0.478 | 0.497 |  | 6.994 | 0.038* | 0.456 | 6.345 | 0.033* | 0.197 |
|  | Age | 2.613 | 0.132 |  | 0.929 | 0.346 |  | 3.749 | 0.101 |  | 2.242 | 0.169 |  |
|  | Site | 4.478 | 0.025* | 0.150 | 0.694 | 0.566 |  | 1.880 | 0.232 |  | 1.402 | 0.295 |  |
|  |  |  |  |  |  |  |  |  |  |  |  |  |  |
| 15-month | AWC | 1.067 | 0.319 |  | 1.645 | 0.211 |  | 0.003 | 0.955 |  | 1.081 | 0.315 |  |
|  | Age | 1.668 | 0.217 |  | 0.440 | 0.513 |  | 0.002 | 0.963 |  | 0.431 | 0.521 |  |
|  | Site | 4.728 | 0.018* | 0.072 | 0.890 | 0.460 |  | 0.745 | 0.550 |  | 1.213 | 0.325 |  |
|  |  |  |  |  |  |  |  |  |  |  |  |  |  |
|  | CVC | 0.003 | 0.959 |  | 1.467 | 0.237 |  | 0.149 | 0.708 |  | 2.205 | 0.158 |  |
|  | Age | 1.289 | 0.275 |  | 0.250 | 0.621 |  | 0.016 | 0.901 |  | 0.066 | 0.801 |  |
|  | Site | 3.773 | 0.036* | 0.144 | 0.749 | 0.533 |  | 0.780 | 0.531 |  | 1.968 | 0.174 |  |
|  |  |  |  |  |  |  |  |  |  |  |  |  |  |
|  | CTC | 0.180 | 0.678 |  | 0.838 | 0.369 |  | 0.006 | 0.939 |  | 0.130 | 0.724 |  |
|  | Age | 1.479 | 0.244 |  | 0.392 | 0.537 |  | 0.001 | 0.972 |  | 0.340 | 0.569 |  |
|  | Site | 4.176 | 0.026* | 0.104 | 0.816 | 0.497 |  | 0.749 | 0.548 |  | 1.513 | 0.252 |  |

| Left Posterior Parieto-Temporal Arcuate Fasciculus | | | | | | | | | | | | | |
| --- | --- | --- | --- | --- | --- | --- | --- | --- | --- | --- | --- | --- | --- |
|  |  | 3-month DTI | | | 6-month DTI | | | 12-month DTI | | | 24-month DTI | | |
| LENA Timepoint | Predictors | F | *p* | *q* | F | *p* | *q* | F | *p* | *q* | F | *p* | *q* |
| 9-month | AWC | 0.029 | 0.867 |  | 0.160 | 0.693 |  | 0.291 | 0.609 |  | 22.506 | 0.001** | 0.013* |
|  | Age | 3.808 | 0.075 |  | 0.102 | 0.753 |  | 0.878 | 0.385 |  | 15.776 | 0.003** | 0.029* |
|  | Site | 0.486 | 0.699 |  | 0.682 | 0.572 |  | 1.012 | 0.418 |  | 4.357 | 0.047* | 0.272 |
|  |  |  |  |  |  |  |  |  |  |  |  |  |  |
|  | CVC | 0.973 | 0.343 |  | 14.286 | 0.001** | 0.012* | 1.160 | 0.323 |  | 3.731 | 0.086 |  |
|  | Age | 4.887 | 0.047* | 0.188 | 0.008 | 0.932 |  | 1.249 | 0.307 |  | 2.239 | 0.169 |  |
|  | Site | 0.435 | 0.732 |  | 0.414 | 0.745 |  | 1.295 | 0.341 |  | 1.819 | 0.217 |  |
|  |  |  |  |  |  |  |  |  |  |  |  |  |  |
|  | CTC | 2.325 | 0.153 |  | 4.854 | 0.038* | 0.461 | 1.485 | 0.269 |  | 23.802 | 0.0009*** | 0.012* |
|  | Age | 5.898 | 0.032* | 0.160 | 0.324 | 0.575 |  | 1.809 | 0.227 |  | 15.482 | 0.003** | 0.020* |
|  | Site | 0.773 | 0.531 |  | 0.367 | 0.778 |  | 1.697 | 0.261 |  | 6.841 | 0.016* | 0.126 |
|  |  |  |  |  |  |  |  |  |  |  |  |  |  |
| 15-month | AWC | 0.043 | 0.839 |  | 0.774 | 0.387 |  | 0.623 | 0.448 |  | 0.0004 | 0.984 |  |
|  | Age | 3.272 | 0.092 |  | 0.114 | 0.739 |  | 0.115 | 0.742 |  | 0.469 | 0.504 |  |
|  | Site | 0.826 | 0.501 |  | 1.107 | 0.365 |  | 1.123 | 0.386 |  | 0.759 | 0.485 |  |
|  |  |  |  |  |  |  |  |  |  |  |  |  |  |
|  | CVC | 0.453 | 0.512 |  | 1.320 | 0.262 |  | 0.720 | 0.416 |  | 2.473 | 0.137 |  |
|  | Age | 3.242 | 0.093 |  | 0.057 | 0.813 |  | 0.073 | 0.793 |  | 0.106 | 0.749 |  |
|  | Site | 0.839 | 0.495 |  | 1.066 | 0.381 |  | 1.448 | 0.287 |  | 0.847 | 0.448 |  |
|  |  |  |  |  |  |  |  |  |  |  |  |  |  |
|  | CTC | 0.354 | 0.561 |  | 0.194 | 0.664 |  | 1.659 | 0.227 |  | 0.125 | 0.729 |  |
|  | Age | 2.538 | 0.134 |  | 0.077 | 0.784 |  | 0.022 | 0.886 |  | 0.442 | 0.516 |  |
|  | Site | 0.934 | 0.450 |  | 1.187 | 0.335 |  | 1.300 | 0.328 |  | 0.835 | 0.453 |  |

| Left Corticospinal Tract | | | | | | | | | | | | | |
| --- | --- | --- | --- | --- | --- | --- | --- | --- | --- | --- | --- | --- | --- |
|  |  | 3-month DTI | | | 6-month DTI | | | 12-month DTI | | | 24-month DTI | | |
| LENA Timepoint | Predictors | F | *p* | *q* | F | *p* | *q* | F | *p* | *q* | F | *p* | *q* |
| 9-month | AWC | 0.046 | 0.835 |  | 2.528 | 0.126 |  | 0.457 | 0.524 |  | 2.130 | 0.178 |  |
|  | Age | 6.847 | 0.024* | 0.225 | 5.006 | 0.036* | 0.428 | 0.146 | 0.716 |  | 9.942 | 0.012* | 0.047* |
|  | Site | 2.293 | 0.135 |  | 2.202 | 0.117 |  | 0.837 | 0.478 |  | 7.776 | 0.011* | 0.132 |
|  |  |  |  |  |  |  |  |  |  |  |  |  |  |
|  | CVC | 0.312 | 0.588 |  | 1.969 | 0.175 |  | 1.469 | 0.271 |  | 0.313 | 0.590 |  |
|  | Age | 7.416 | 0.020* | 0.188 | 2.997 | 0.097 |  | 0.076 | 0.793 |  | 6.341 | 0.033* | 0.197 |
|  | Site | 2.289 | 0.135 |  | 2.001 | 0.143 |  | 1.153 | 0.377 |  | 7.135 | 0.014* | 0.167 |
|  |  |  |  |  |  |  |  |  |  |  |  |  |  |
|  | CTC | 0.130 | 0.726 |  | 1.242 | 0.277 |  | 2.570 | 0.160 |  | 0.329 | 0.580 |  |
|  | Age | 6.128 | 0.031* | 0.160 | 3.976 | 0.059 |  | 0.001 | 0.976 |  | 6.669 | 0.030* | 0.099 |
|  | Site | 2.409 | 0.122 |  | 1.909 | 0.158 |  | 0.785 | 0.498 |  | 6.108 | 0.021* | 0.126 |
|  |  |  |  |  |  |  |  |  |  |  |  |  |  |
| 15-month | AWC | 0.074 | 0.791 |  | 4.192 | 0.051 |  | 0.008 | 0.931 |  | 0.054 | 0.820 |  |
|  | Age | 10.495 | 0.007** | 0.084 | 5.301 | 0.030* | 0.360 | 0.567 | 0.469 |  | 7.627 | 0.015* | 0.144 |
|  | Site | 2.577 | 0.102 |  | 1.273 | 0.305 |  | 1.311 | 0.324 |  | 5.447 | 0.017* | 0.102 |
|  |  |  |  |  |  |  |  |  |  |  |  |  |  |
|  | CVC | 0.137 | 0.717 |  | 1.011 | 0.324 |  | 1.565 | 0.239 |  | 1.197 | 0.291 |  |
|  | Age | 9.900 | 0.008** | 0.084 | 3.669 | 0.067 |  | 1.050 | 0.330 |  | 6.237 | 0.025* | 0.246 |
|  | Site | 2.630 | 0.098 |  | 1.584 | 0.218 |  | 1.729 | 0.224 |  | 6.527 | 0.009** | 0.054 |
|  |  |  |  |  |  |  |  |  |  |  |  |  |  |
|  | CTC | 0.207 | 0.658 |  | 1.257 | 0.273 |  | 0.321 | 0.584 |  | 1.478 | 0.243 |  |
|  | Age | 8.743 | 0.012* | 0.144 | 4.309 | 0.048* | 0.390 | 0.756 | 0.405 |  | 7.872 | 0.013* | 0.108 |
|  | Site | 2.694 | 0.093 |  | 1.487 | 0.242 |  | 1.267 | 0.338 |  | 6.682 | 0.008** | 0.048* |

| Left Inferior Frontal-Occipital Fasciculus | | | | | | | | | | | | | |
| --- | --- | --- | --- | --- | --- | --- | --- | --- | --- | --- | --- | --- | --- |
|  |  | 3-month DTI | | | 6-month DTI | | | 12-month DTI | | | 24-month DTI | | |
| LENA Timepoint | Predictors | F | *p* | *q* | F | *p* | *q* | F | *p* | *q* | F | *p* | *q* |
| 9-month | AWC | 0.040 | 0.845 |  | 0.017 | 0.898 |  | 0.015 | 0.906 |  | 1.506 | 0.251 |  |
|  | Age | 0.001 | 0.981 |  | 0.072 | 0.791 |  | 0.0001 | 0.991 |  | 0.592 | 0.461 |  |
|  | Site | 0.191 | 0.901 |  | 0.748 | 0.535 |  | 0.889 | 0.459 |  | 0.260 | 0.777 |  |
|  |  |  |  |  |  |  |  |  |  |  |  |  |  |
|  | CVC | 0.049 | 0.828 |  | 3.579 | 0.072 |  | 0.170 | 0.694 |  | 0.050 | 0.829 |  |
|  | Age | 0.020 | 0.923 |  | 0.021 | 0.885 |  | 0.002 | 0.964 |  | 0.057 | 0.816 |  |
|  | Site | 0.246 | 0.863 |  | 0.372 | 0.774 |  | 0.926 | 0.446 |  | 0.273 | 0.767 |  |
|  |  |  |  |  |  |  |  |  |  |  |  |  |  |
|  | CTC | 0.160 | 0.696 |  | 1.095 | 0.307 |  | 0.213 | 0.661 |  | 0.624 | 0.450 |  |
|  | Age | 0.019 | 0.894 |  | 0.219 | 0.644 |  | 0.018 | 0.897 |  | 0.303 | 0.596 |  |
|  | Site | 0.268 | 0.847 |  | 0.512 | 0.679 |  | 1.007 | 0.420 |  | 0.145 | 0.867 |  |
|  |  |  |  |  |  |  |  |  |  |  |  |  |  |
| 15-month | AWC | 0.722 | 0.410 |  | 0.230 | 0.636 |  | 0.464 | 0.511 |  | 0.022 | 0.886 |  |
|  | Age | 0.388 | 0.544 |  | 0.143 | 0.708 |  | 0.027 | 0.874 |  | 0.027 | 0.871 |  |
|  | Site | 0.187 | 0.904 |  | 0.681 | 0.572 |  | 0.684 | 0.582 |  | 0.658 | 0.533 |  |
|  |  |  |  |  |  |  |  |  |  |  |  |  |  |
|  | CVC | 1.839 | 0.197 |  | 0.479 | 0.495 |  | 1.197 | 0.300 |  | 2.984 | 0.106 |  |
|  | Age | 0.254 | 0.622 |  | 0.078 | 0.783 |  | 0.084 | 0.778 |  | 1.004 | 0.333 |  |
|  | Site | 0.200 | 0.895 |  | 0.477 | 0.701 |  | 0.961 | 0.448 |  | 1.515 | 0.254 |  |
|  |  |  |  |  |  |  |  |  |  |  |  |  |  |
|  | CTC | 0.084 | 0.776 |  | 0.012 | 0.915 |  | 1.022 | 0.336 |  | 2.082 | 0.171 |  |
|  | Age | 0.177 | 0.681 |  | 0.075 | 0.786 |  | 0.099 | 0.759 |  | 0.252 | 0.624 |  |
|  | Site | 0.111 | 0.953 |  | 0.641 | 0.596 |  | 0.809 | 0.517 |  | 1.238 | 0.320 |  |

| Left Inferior Longitudinal Fasciculus | | | | | | | | | | | | | |
| --- | --- | --- | --- | --- | --- | --- | --- | --- | --- | --- | --- | --- | --- |
|  |  | 3-month DTI | | | 6-month DTI | | | 12-month DTI | | | 24-month DTI | | |
| LENA Timepoint | Predictors | F | *p* | *q* | F | *p* | *q* | F | *p* | *q* | F | *p* | *q* |
| 9-month | AWC | 0.002 | 0.968 |  | 0.268 | 0.610 |  | 0.630 | 0.458 |  | 4.169 | 0.072 |  |
|  | Age | 0.438 | 0.521 |  | 0.188 | 0.669 |  | 0.072 | 0.798 |  | 4.255 | 0.069 |  |
|  | Site | 0.528 | 0.671 |  | 1.114 | 0.365 |  | 0.472 | 0.645 |  | 1.206 | 0.343 |  |
|  |  |  |  |  |  |  |  |  |  |  |  |  |  |
|  | CVC | 0.200 | 0.663 |  | 5.097 | 0.034* | 0.205 | 0.046 | 0.837 |  | 0.003 | 0.956 |  |
|  | Age | 0.581 | 0.461 |  | 0.015 | 0.904 |  | 0.099 | 0.763 |  | 1.195 | 0.303 |  |
|  | Site | 0.555 | 0.655 |  | 0.621 | 0.609 |  | 0.571 | 0.593 |  | 0.510 | 0.617 |  |
|  |  |  |  |  |  |  |  |  |  |  |  |  |  |
|  | CTC | 0.169 | 0.688 |  | 2.721 | 0.113 |  | 0.169 | 0.695 |  | 1.874 | 0.204 |  |
|  | Age | 0.556 | 0.470 |  | 0.325 | 0.575 |  | 0.177 | 0.688 |  | 2.686 | 0.136 |  |
|  | Site | 0.612 | 0.620 |  | 0.772 | 0.522 |  | 0.607 | 0.575 |  | 1.005 | 0.404 |  |
|  |  |  |  |  |  |  |  |  |  |  |  |  |  |
| 15-month | AWC | 0.006 | 0.941 |  | 0.056 | 0.815 |  | 0.008 | 0.930 |  | 0.091 | 0.767 |  |
|  | Age | 1.615 | 0.225 |  | 0.037 | 0.850 |  | 0.030 | 0.866 |  | 2.787 | 0.116 |  |
|  | Site | 1.350 | 0.299 |  | 1.446 | 0.253 |  | 0.502 | 0.690 |  | 0.446 | 0.648 |  |
|  |  |  |  |  |  |  |  |  |  |  |  |  |  |
|  | CVC | 5.315 | 0.037* | 0.444 | 1.715 | 0.202 |  | 0.089 | 0.772 |  | 1.909 | 0.187 |  |
|  | Age | 2.010 | 0.178 |  | 0.037 | 0.849 |  | 0.011 | 0.918 |  | 1.850 | 0.194 |  |
|  | Site | 1.363 | 0.295 |  | 1.082 | 0.375 |  | 0.533 | 0.670 |  | 0.433 | 0.656 |  |
|  |  |  |  |  |  |  |  |  |  |  |  |  |  |
|  | CTC | 0.615 | 0.446 |  | 0.436 | 0.515 |  | 0.003 | 0.960 |  | 0.364 | 0.555 |  |
|  | Age | 1.087 | 0.315 |  | 0.010 | 0.923 |  | 0.029 | 0.868 |  | 2.680 | 0.122 |  |
|  | Site | 1.441 | 0.273 |  | 1.453 | 0.251 |  | 0.505 | 0.688 |  | 0.560 | 0.583 |  |

| Splenium of the Corpus Callosum | | | | | | | | | | | | | |
| --- | --- | --- | --- | --- | --- | --- | --- | --- | --- | --- | --- | --- | --- |
|  |  | 3-month DTI | | | 6-month DTI | | | 12-month DTI | | | 24-month DTI | | |
| LENA Timepoint | Predictors | F | *p* | *q* | F | *p* | *q* | F | *p* | *q* | F | *p* | *q* |
| 9-month | AWC | 0.066 | 0.802 |  | 1.938 | 0.178 |  | 0.093 | 0.7706 |  | 0.379 | 0.554 |  |
|  | Age | 2.348 | 0.151 |  | 0.283 | 0.600 |  | 1.002 | 0.356 |  | 2.903 | 0.123 |  |
|  | Site | 0.607 | 0.623 |  | 1.179 | 0.340 |  | 0.835 | 0.475 |  | 1.783 | 0.223 |  |
|  |  |  |  |  |  |  |  |  |  |  |  |  |  |
|  | CVC | 0.260 | 0.619 |  | 0.523 | 0.477 |  | 0.001 | 0.981 |  | 0.296 | 0.600 |  |
|  | Age | 2.717 | 0.125 |  | 0.066 | 0.800 |  | 0.939 | 0.370 |  | 2.395 | 0.156 |  |
|  | Site | 0.586 | 0.636 |  | 0.651 | 0.591 |  | 0.829 | 0.481 |  | 1.483 | 0.278 |  |
|  |  |  |  |  |  |  |  |  |  |  |  |  |  |
|  | CTC | 0.001 | 0.891 |  | 0.127 | 0.725 |  | 0.052 | 0.827 |  | 0.008 | 0.932 |  |
|  | Age | 2.285 | 0.157 |  | 0.055 | 0.817 |  | 0.790 | 0.408 |  | 2.167 | 0.175 |  |
|  | Site | 0.585 | 0.636 |  | 0.962 | 0.428 |  | 0.679 | 0.542 |  | 1.520 | 0.270 |  |
|  |  |  |  |  |  |  |  |  |  |  |  |  |  |
| 15-month | AWC | 0.010 | 0.923 |  | 1.087 | 0.307 |  | 0.019 | 0.894 |  | 0.002 | 0.969 |  |
|  | Age | 1.127 | 0.306 |  | 0.103 | 0.751 |  | 0.315 | 0.587 |  | 0.545 | 0.472 |  |
|  | Site | 0.617 | 0.616 |  | 1.306 | 0.294 |  | 0.920 | 0.467 |  | 1.341 | 0.291 |  |
|  |  |  |  |  |  |  |  |  |  |  |  |  |  |
|  | CVC | 0.934 | 0.350 |  | 2.875 | 0.102 |  | 2.618 | 0.137 |  | 6.651 | 0.021* | 0.252 |
|  | Age | 1.175 | 0.297 |  | 0.009 | 0.927 |  | 0.113 | 0.743 |  | 0.048 | 0.830 |  |
|  | Site | 0.897 | 0.467 |  | 0.990 | 0.414 |  | 0.677 | 0.586 |  | 1.066 | 0.369 |  |
|  |  |  |  |  |  |  |  |  |  |  |  |  |  |
|  | CTC | 1.042 | 0.325 |  | 0.014 | 0.908 |  | 1.167 | 0.305 |  | 3.769 | 0.071 |  |
|  | Age | 0.652 | 0.433 |  | 0.015 | 0.905 |  | 0.122 | 0.734 |  | 0.502 | 0.490 |  |
|  | Site | 0.947 | 0.444 |  | 0.994 | 0.412 |  | 0.948 | 0.454 |  | 1.704 | 0.215 |  |

| Left Uncinate Fasciculus | | | | | | | | | | | | | |
| --- | --- | --- | --- | --- | --- | --- | --- | --- | --- | --- | --- | --- | --- |
|  |  | 3-month DTI | | | 6-month DTI | | | 12-month DTI | | | 24-month DTI | | |
| LENA Timepoint | Predictors | F | *p* | *q* | F | *p* | *q* | F | *p* | *q* | F | *p* | *q* |
| 9-month | AWC | 0.208 | 0.657 |  | 0.157 | 0.696 |  | 0.058 | 0.818 |  | 0.658 | 0.438 |  |
|  | Age | 0.039 | 0.848 |  | 0.413 | 0.527 |  | 0.012 | 0.917 |  | 0.052 | 0.825 |  |
|  | Site | 0.605 | 0.625 |  | 0.322 | 0.820 |  | 0.916 | 0.450 |  | 0.535 | 0.603 |  |
|  |  |  |  |  |  |  |  |  |  |  |  |  |  |
|  | CVC | 1.826 | 0.202 |  | 0.268 | 0.620 |  | 0.217 | 0.658 |  | 0.189 | 0.674 |  |
|  | Age | 0.365 | 0.557 |  | 0.507 | 0.484 |  | 0.026 | 0.878 |  | 0.345 | 0.572 |  |
|  | Site | 0.385 | 0.766 |  | 0.447 | 0.722 |  | 0.915 | 0.450 |  | 0.691 | 0.526 |  |
|  |  |  |  |  |  |  |  |  |  |  |  |  |  |
|  | CTC | 0.050 | 0.827 |  | 0.088 | 0.770 |  | 0.371 | 0.565 |  | 0.915 | 0.364 |  |
|  | Age | 0.029 | 0.869 |  | 0.639 | 0.433 |  | 0.078 | 0.790 |  | 0.047 | 0.834 |  |
|  | Site | 0.557 | 0.653 |  | 0.418 | 0.742 |  | 1.022 | 0.415 |  | 0.734 | 0.507 |  |
|  |  |  |  |  |  |  |  |  |  |  |  |  |  |
| 15-month | AWC | 0.348 | 0.565 |  | 0.079 | 0.781 |  | 0.456 | 0.515 |  | 0.592 | 0.454 |  |
|  | Age | 0.040 | 0.845 |  | 0.976 | 0.333 |  | 0.142 | 0.714 |  | 0.315 | 0.584 |  |
|  | Site | 0.900 | 0.466 |  | 0.313 | 0.816 |  | 0.265 | 0.849 |  | 1.615 | 0.234 |  |
|  |  |  |  |  |  |  |  |  |  |  |  |  |  |
|  | CVC | 0.701 | 0.416 |  | 0.749 | 0.395 |  | 5.777 | 0.037* | 0.414 | 0.665 | 0.428 |  |
|  | Age | 0.026 | 0.875 |  | 1.196 | 0.285 |  | 0.011 | 0.920 |  | 0.805 | 0.385 |  |
|  | Site | 1.031 | 0.409 |  | 0.497 | 0.688 |  | 0.937 | 0.459 |  | 1.688 | 0.221 |  |
|  |  |  |  |  |  |  |  |  |  |  |  |  |  |
|  | CTC | 2.102 | 0.169 |  | 0.001 | 0.981 |  | 2.286 | 0.162 |  | 1.323 | 0.269 |  |
|  | Age | 0.270 | 0.611 |  | 1.042 | 0.317 |  | 0.019 | 0.893 |  | 0.690 | 0.420 |  |
|  | Site | 1.335 | 0.303 |  | 0.351 | 0.789 |  | 0.464 | 0.714 |  | 2.077 | 0.162 |  |

| Right Anterior Fronto-Parietal Arcuate Fasciculus | | | | | | | | | | | | | |
| --- | --- | --- | --- | --- | --- | --- | --- | --- | --- | --- | --- | --- | --- |
|  |  | 3-month DTI | | | 6-month DTI | | | 12-month DTI | | | 24-month DTI | | |
| LENA Timepoint | Predictors | F | *p* | *q* | F | *p* | *q* | F | *p* | *q* | F | *p* | *q* |
| 9-month | AWC | 0.018 | 0.896 |  | 1.538 | 0.228 |  | 0.146 | 0.716 |  | 0.187 | 0.676 |  |
|  | Age | 5.406 | 0.038* | 0.225 | 1.606 | 0.218 |  | 0.150 | 0.712 |  | 0.002 | 0.964 |  |
|  | Site | 8.358 | 0.003** | 0.036* | 1.126 | 0.360 |  | 0.893 | 0.458 |  | 2.520 | 0.135 |  |
|  |  |  |  |  |  |  |  |  |  |  |  |  |  |
|  | CVC | 0.0003 | 0.987 |  | 0.0003 | 0.987 |  | 0.184 | 0.683 |  | 0.619 | 0.452 |  |
|  | Age | 5.007 | 0.045* | 0.188 | 0.903 | 0.352 |  | 0.108 | 0.754 |  | 0.097 | 0.763 |  |
|  | Site | 8.906 | 0.002** | 0.024* | 1.036 | 0.396 |  | 1.010 | 0.419 |  | 2.595 | 0.129 |  |
|  |  |  |  |  |  |  |  |  |  |  |  |  |  |
|  | CTC | 0.042 | 0.842 |  | 0.266 | 0.611 |  | 0.424 | 0.539 |  | 0.292 | 0.602 |  |
|  | Age | 5.307 | 0.040* | 0.160 | 1.056 | 0.315 |  | 0.037 | 0.854 |  | 0.001 | 0.981 |  |
|  | Site | 8.483 | 0.003** | 0.036* | 0.909 | 0.453 |  | 0.768 | 0.505 |  | 2.760 | 0.116 |  |
|  |  |  |  |  |  |  |  |  |  |  |  |  |  |
| 15-month | AWC | 0.037 | 0.850 |  | 1.042 | 0.317 |  | 0.045 | 0.837 |  | 2.066 | 0.171 |  |
|  | Age | 3.794 | 0.072 |  | 1.213 | 0.281 |  | 0.853 | 0.377 |  | 0.644 | 0.435 |  |
|  | Site | 7.647 | 0.003** | 0.036* | 1.671 | 0.199 |  | 1.518 | 0.269 |  | 0.824 | 0.458 |  |
|  |  |  |  |  |  |  |  |  |  |  |  |  |  |
|  | CVC | 0.949 | 0.347 |  | 0.649 | 0.428 |  | 0.403 | 0.540 |  | 0.184 | 0.674 |  |
|  | Age | 3.875 | 0.069 |  | 0.937 | 0.342 |  | 0.698 | 0.423 |  | 0.343 | 0.567 |  |
|  | Site | 8.543 | 0.002** | 0.024* | 1.829 | 0.168 |  | 1.547 | 0.263 |  | 1.065 | 0.369 |  |
|  |  |  |  |  |  |  |  |  |  |  |  |  |  |
|  | CTC | 0.119 | 0.735 |  | 0.815 | 0.375 |  | 0.028 | 0.871 |  | 0.014 | 0.906 |  |
|  | Age | 3.178 | 0.096 |  | 1.220 | 0.280 |  | 0.793 | 0.394 |  | 0.514 | 0.485 |  |
|  | Site | 8.630 | 0.002** | 0.024* | 1.710 | 0.191 |  | 1.422 | 0.293 |  | 0.901 | 0.427 |  |

| Right Corticospinal Tract | | | | | | | | | | | | | |
| --- | --- | --- | --- | --- | --- | --- | --- | --- | --- | --- | --- | --- | --- |
|  |  | 3-month DTI | | | 6-month DTI | | | 12-month DTI | | | 24-month DTI | | |
| LENA Timepoint | Predictors | F | *p* | *q* | F | *p* | *q* | F | *p* | *q* | F | *p* | *q* |
| 9-month | AWC | 0.844 | 0.378 |  | 0.234 | 0.633 |  | 1.164 | 0.322 |  | 2.213 | 0.171 |  |
|  | Age | 4.117 | 0.067 |  | 0.025 | 0.875 |  | 0.065 | 0.808 |  | 5.271 | 0.047* | 0.142 |
|  | Site | 3.381 | 0.058 |  | 3.034 | 0.051 |  | 0.936 | 0.443 |  | 3.680 | 0.068 |  |
|  |  |  |  |  |  |  |  |  |  |  |  |  |  |
|  | CVC | 0.253 | 0.625 |  | 0.331 | 0.571 |  | 4.464 | 0.079 |  | 0.787 | 0.398 |  |
|  | Age | 3.376 | 0.093 |  | 0.0003 | 0.986 |  | 0.094 | 0.770 |  | 3.136 | 0.110 |  |
|  | Site | 4.033 | 0.037* | 0.148 | 2.878 | 0.059 |  | 0.977 | 0.429 |  | 4.083 | 0.055 |  |
|  |  |  |  |  |  |  |  |  |  |  |  |  |  |
|  | CTC | 0.002 | 0.964 |  | 0.123 | 0.729 |  | 0.183 | 0.684 |  | 3.490 | 0.095 |  |
|  | Age | 3.653 | 0.082 |  | 0.0002 | 0.990 |  | 0.059 | 0.816 |  | 6.325 | 0.033* | 0.099 |
|  | Site | 3.634 | 0.048* | 0.192 | 3.297 | 0.039* | 0.473 | 0.543 | 0.607 |  | 4.358 | 0.048* | 0.190 |
|  |  |  |  |  |  |  |  |  |  |  |  |  |  |
| 15-month | AWC | 0.870 | 0.368 |  | 2.099 | 0.160 |  | 4.454 | 0.061 |  | 0.261 | 0.617 |  |
|  | Age | 7.625 | 0.016* | 0.096 | 0.112 | 0.741 |  | 2.767 | 0.127 |  | 6.313 | 0.024* | 0.144 |
|  | Site | 5.352 | 0.013* | 0.072 | 1.052 | 0.387 |  | 1.146 | 0.378 |  | 6.810 | 0.008** | 0.096 |
|  |  |  |  |  |  |  |  |  |  |  |  |  |  |
|  | CVC | 0.255 | 0.622 |  | 2.647 | 0.116 |  | 0.040 | 0.845 |  | 4.157 | 0.060 |  |
|  | Age | 7.787 | 0.015* | 0.096 | 0.025 | 0.877 |  | 2.367 | 0.155 |  | 5.016 | 0.041* | 0.246 |
|  | Site | 6.388 | 0.007** | 0.042* | 0.890 | 0.460 |  | 0.409 | 0.750 |  | 9.858 | 0.002** | 0.024* |
|  |  |  |  |  |  |  |  |  |  |  |  |  |  |
|  | CTC | 2.236 | 0.159 |  | 1.461 | 0.238 |  | 2.884 | 0.120 |  | 3.405 | 0.085 |  |
|  | Age | 5.303 | 0.038* | 0.228 | 0.113 | 0.739 |  | 1.902 | 0.198 |  | 7.095 | 0.018* | 0.108 |
|  | Site | 7.238 | 0.004** | 0.024* | 1.199 | 0.331 |  | 0.895 | 0.477 |  | 9.722 | 0.002** | 0.024* |

| Right Inferior Frontal-Occipital Fasciculus | | | | | | | | | | | | | |
| --- | --- | --- | --- | --- | --- | --- | --- | --- | --- | --- | --- | --- | --- |
|  |  | 3-month DTI | | | 6-month DTI | | | 12-month DTI | | | 24-month DTI | | |
| LENA Timepoint | Predictors | F | *p* | *q* | F | *p* | *q* | F | *p* | *q* | F | *p* | *q* |
| 9-month | AWC | 0.156 | 0.700 |  | 0.515 | 0.480 |  | 0.0000 | 0.999 |  | 1.441 | 0.261 |  |
|  | Age | 0.062 | 0.808 |  | 0.962 | 0.337 |  | 0.015 | 0.906 |  | 0.089 | 0.772 |  |
|  | Site | 0.426 | 0.738 |  | 0.698 | 0.563 |  | 2.301 | 0.181 |  | 0.484 | 0.631 |  |
|  |  |  |  |  |  |  |  |  |  |  |  |  |  |
|  | CVC | 1.028 | 0.331 |  | 0.041 | 0.842 |  | 0.096 | 0.767 |  | 0.007 | 0.937 |  |
|  | Age | 0.307 | 0.590 |  | 1.437 | 0.243 |  | 0.020 | 0.892 |  | 0.049 | 0.830 |  |
|  | Site | 0.532 | 0.669 |  | 0.756 | 0.531 |  | 2.455 | 0.166 |  | 0.343 | 0.719 |  |
|  |  |  |  |  |  |  |  |  |  |  |  |  |  |
|  | CTC | 0.007 | 0.936 |  | 0.734 | 0.401 |  | 0.094 | 0.770 |  | 1.207 | 0.300 |  |
|  | Age | 0.062 | 0.807 |  | 1.134 | 0.299 |  | 0.038 | 0.852 |  | 0.047 | 0.834 |  |
|  | Site | 0.423 | 0.740 |  | 0.770 | 0.523 |  | 2.468 | 0.165 |  | 0.281 | 0.762 |  |
|  |  |  |  |  |  |  |  |  |  |  |  |  |  |
| 15-month | AWC | 0.0002 | 0.990 |  | 0.029 | 0.866 |  | 1.920 | 0.196 |  | 0.276 | 0.607 |  |
|  | Age | 0.252 | 0.623 |  | 3.673 | 0.067 |  | 0.419 | 0.532 |  | 0.355 | 0.560 |  |
|  | Site | 0.448 | 0.722 |  | 1.522 | 0.233 |  | 0.698 | 0.574 |  | 0.348 | 0.712 |  |
|  |  |  |  |  |  |  |  |  |  |  |  |  |  |
|  | CVC | 0.687 | 0.421 |  | 0.221 | 0.642 |  | 2.768 | 0.127 |  | 0.115 | 0.740 |  |
|  | Age | 0.298 | 0.594 |  | 3.981 | 0.057 |  | 0.268 | 0.616 |  | 0.413 | 0.530 |  |
|  | Site | 0.563 | 0.649 |  | 1.598 | 0.215 |  | 0.527 | 0.674 |  | 0.279 | 0.760 |  |
|  |  |  |  |  |  |  |  |  |  |  |  |  |  |
|  | CTC | 0.456 | 0.510 |  | 0.0001 | 0.991 |  | 2.746 | 0.129 |  | 0.106 | 0.749 |  |
|  | Age | 0.457 | 0.510 |  | 3.716 | 0.065 |  | 0.194 | 0.669 |  | 0.309 | 0.587 |  |
|  | Site | 0.417 | 0.744 |  | 1.500 | 0.239 |  | 0.786 | 0.529 |  | 0.293 | 0.750 |  |

| Right Inferior Longitudinal Fasciculus | | | | | | | | | | | | | |
| --- | --- | --- | --- | --- | --- | --- | --- | --- | --- | --- | --- | --- | --- |
|  |  | 3-month DTI | | | 6-month DTI | | | 12-month DTI | | | 24-month DTI | | |
| LENA Timepoint | Predictors | F | *p* | *q* | F | *p* | *q* | F | *p* | *q* | F | *p* | *q* |
| 9-month | AWC | 0.314 | 0.586 |  | 0.141 | 0.711 |  | 0.063 | 0.811 |  | 0.956 | 0.354 |  |
|  | Age | 0.033 | 0.859 |  | 0.007 | 0.937 |  | 0.031 | 0.865 |  | 13.794 | 0.005** | 0.029* |
|  | Site | 0.309 | 0.818 |  | 0.720 | 0.551 |  | 0.202 | 0.822 |  | 1.222 | 0.339 |  |
|  |  |  |  |  |  |  |  |  |  |  |  |  |  |
|  | CVC | 0.277 | 0.609 |  | 0.850 | 0.367 |  | 0.120 | 0.752 |  | 0.468 | 0.511 |  |
|  | Age | 0.129 | 0.725 |  | 0.011 | 0.917 |  | 0.019 | 0.896 |  | 12.890 | 0.006** | 0.070 |
|  | Site | 0.211 | 0.887 |  | 0.509 | 0.680 |  | 0.183 | 0.837 |  | 1.108 | 0.371 |  |
|  |  |  |  |  |  |  |  |  |  |  |  |  |  |
|  | CTC | 0.316 | 0.585 |  | 0.413 | 0.527 |  | 0.282 | 0.614 |  | 2.728 | 0.133 |  |
|  | Age | 0.127 | 0.728 |  | 0.007 | 0.936 |  | 0.001 | 0.982 |  | 18.369 | 0.002** | 0.020* |
|  | Site | 0.253 | 0.858 |  | 0.595 | 0.625 |  | 0.243 | 0.792 |  | 1.864 | 0.210 |  |
|  |  |  |  |  |  |  |  |  |  |  |  |  |  |
| 15-month | AWC | 0.234 | 0.636 |  | 1.065 | 0.312 |  | 0.339 | 0.573 |  | 2.121 | 0.166 |  |
|  | Age | 0.259 | 0.619 |  | 0.128 | 0.723 |  | 0.205 | 0.661 |  | 5.311 | 0.036* | 0.144 |
|  | Site | 0.725 | 0.554 |  | 1.134 | 0.354 |  | 0.384 | 0.767 |  | 1.362 | 0.286 |  |
|  |  |  |  |  |  |  |  |  |  |  |  |  |  |
|  | CVC | 1.007 | 0.333 |  | 0.012 | 0.913 |  | 0.550 | 0.475 |  | 1.601 | 0.225 |  |
|  | Age | 0.190 | 0.669 |  | 0.036 | 0.851 |  | 0.435 | 0.524 |  | 3.391 | 0.085 |  |
|  | Site | 0.543 | 0.661 |  | 1.011 | 0.404 |  | 0.639 | 0.607 |  | 1.688 | 0.218 |  |
|  |  |  |  |  |  |  |  |  |  |  |  |  |  |
|  | CTC | 0.187 | 0.672 |  | 0.614 | 0.441 |  | 0.002 | 0.966 |  | 0.318 | 0.581 |  |
|  | Age | 0.105 | 0.751 |  | 0.117 | 0.735 |  | 0.242 | 0.633 |  | 4.411 | 0.053 |  |
|  | Site | 0.619 | 0.614 |  | 1.113 | 0.363 |  | 0.477 | 0.706 |  | 1.698 | 0.216 |  |

| Right Uncinate Fasciculus | | | | | | | | | | | | | |
| --- | --- | --- | --- | --- | --- | --- | --- | --- | --- | --- | --- | --- | --- |
|  |  | 3-month DTI | | | 6-month DTI | | | 12-month DTI | | | 24-month DTI | | |
| LENA Timepoint | Predictors | F | *p* | *q* | F | *p* | *q* | F | *p* | *q* | F | *p* | *q* |
| 9-month | AWC | 0.410 | 0.534 |  | 0.841 | 0.369 |  | 0.109 | 0.752 |  | 0.864 | 0.377 |  |
|  | Age | 2.595 | 0.133 |  | 0.041 | 0.842 |  | 1.211 | 0.313 |  | 0.401 | 0.543 |  |
|  | Site | 0.945 | 0.449 |  | 0.345 | 0.793 |  | 3.578 | 0.095 |  | 0.167 | 0.849 |  |
|  |  |  |  |  |  |  |  |  |  |  |  |  |  |
|  | CVC | 0.346 | 0.568 |  | 1.420 | 0.246 |  | 0.251 | 0.634 |  | 0.211 | 0.657 |  |
|  | Age | 2.069 | 0.176 |  | 0.321 | 0.577 |  | 1.239 | 0.308 |  | 0.080 | 0.783 |  |
|  | Site | 0.912 | 0.464 |  | 0.484 | 0.697 |  | 3.636 | 0.092 |  | 0.168 | 0.848 |  |
|  |  |  |  |  |  |  |  |  |  |  |  |  |  |
|  | CTC | 0.006 | 0.941 |  | 1.981 | 0.173 |  | 0.0002 | 0.988 |  | 0.506 | 0.495 |  |
|  | Age | 2.463 | 0.143 |  | 0.060 | 0.809 |  | 1.074 | 0.340 |  | 0.266 | 0.619 |  |
|  | Site | 0.749 | 0.544 |  | 0.451 | 0.719 |  | 3.429 | 0.102 |  | 0.223 | 0.804 |  |
|  |  |  |  |  |  |  |  |  |  |  |  |  |  |
| 15-month | AWC | 1.120 | 0.308 |  | 0.337 | 0.567 |  | 0.673 | 0.431 |  | 0.010 | 0.921 |  |
|  | Age | 1.829 | 0.198 |  | 0.377 | 0.545 |  | 0.052 | 0.825 |  | 1.211 | 0.288 |  |
|  | Site | 1.043 | 0.404 |  | 0.860 | 0.475 |  | 1.261 | 0.340 |  | 0.211 | 0.812 |  |
|  |  |  |  |  |  |  |  |  |  |  |  |  |  |
|  | CVC | 0.008 | 0.929 |  | 0.007 | 0.934 |  | 3.086 | 0.110 |  | 0.264 | 0.615 |  |
|  | Age | 1.425 | 0.252 |  | 0.507 | 0.483 |  | 0.223 | 0.647 |  | 0.893 | 0.360 |  |
|  | Site | 0.640 | 0.602 |  | 0.824 | 0.493 |  | 2.230 | 0.147 |  | 0.226 | 0.801 |  |
|  |  |  |  |  |  |  |  |  |  |  |  |  |  |
|  | CTC | 0.029 | 0.868 |  | 0.840 | 0.368 |  | 2.360 | 0.156 |  | 0.338 | 0.570 |  |
|  | Age | 1.426 | 0.252 |  | 0.278 | 0.603 |  | 0.241 | 0.634 |  | 1.152 | 0.300 |  |
|  | Site | 0.682 | 0.578 |  | 0.938 | 0.437 |  | 1.878 | 0.197 |  | 0.319 | 0.732 |  |

The following table contains site-wise breakdown of LENA data collection at 9- and 15-month time points.

| Data collection site | Home language data at 9-months (*n*) | | | | | Home language data at 15-months (*n*) | | | | |
| --- | --- | --- | --- | --- | --- | --- | --- | --- | --- | --- |
|  | 3-mo | 6-mo | 12-mo | 24-mo | **Total** | 3-mo | 6-mo | 12-mo | 24-mo | **Total** |
| UNC | 3 | 12 | 6 | 6 | 27 | 4 | 14 | 7 | 9 | 34 |
| PHI | 3 | 5 | 0 | 0 | 8 | 4 | 4 | 2 | 0 | 10 |
| SEA | 5 | 3 | 2 | 2 | 12 | 5 | 3 | 2 | 2 | 12 |
| STL | 7 | 8 | 3 | 6 | 24 | 7 | 10 | 5 | 9 | 31 |
| Total | 18 | 28 | 11 | 14 |  | 20 | 31 | 16 | 20 |  |
